# Safety of Plasma Rich in Growth Factors (PRGF) as additive to healthy human sperm samples: a pilot study

**DOI:** 10.5935/1518-0557.20230075

**Published:** 2024

**Authors:** Fernando Quintana, Alberto Vendrell, Silvia Perez-Fernandez, Maria de la Fuente, Aitana Merino-Pérez, Marcos Ferrando, Roberto Matorras

**Affiliations:** 1 Instituto Valenciano de Infertilidad (IVI), IVIRMA, Leioa 48940, Spain; 2 Biocruces Health Research Institute, Baracaldo 48903, Spain; 3 Cruces University Hospital, Obstetrics and Gynecology Department, Baracaldo 48903, Spain; 4 BTI Biotechnology Institute, Vitoria 01005, Spain; 5 IVI Foundation, Health Research Institute La Fe, Valencia, Spain

**Keywords:** donors, motility, plasma rich in growth factors (PRGF), spermiogram, sperm vitality, thawed

## Abstract

**Objective:**

The aim of our study was to assess if the addition of PRGF to healthy human sperm affects its motility and vitality.

**Methods:**

This was a prospective study, with 44 sperm donors on whom sperm analysis was performed. Nine mL of blood was collected and PRGF was obtained using PRGF-Endoret^®^ technology. The influence of different dilutions of PRGF (5%, 10%, 20%, 40%) applied to 15 sperm donors was compared, and sperm motility was assessed after 30 minutes. In the second part of the study, 29 sperm donors were studied to analyze the influence of 20% dilution of PRGF at 15, 30 and 45 minutes in fresh and thawed sperm samples. Motility was assessed after the addition of PRGF and after analysis each aliquot was frozen. After thawing, concentration and motility were assessed at the same time periods.

**Results:**

There were no differences in sperm motility in fresh samples between dilutions of PRGF when assessed 30 minutes after administration, nor between them, nor when compared to the control group immediately prior to treatment. No trend was observed between motility and PRGF dilution in linear regression analysis. There were no significant differences in thawed samples.

**Conclusions:**

The administration of 20% PRGF dilution had no effect on sperm motility compared to samples without PRGF. In addition, there was no change in sperm vitality when comparing samples with and without PRGF. More studies focusing on subnormal sperm samples, analyzing different PRGF concentrations and increasing the number of study variables are needed.

## INTRODUCTION

Male factor infertility represents one of the main indications for Assisted Reproductive Techniques (ART). Traditionally, moderate to severe male factors are subjected to IVF/ICSI while IUI is employed in light male factors and in cases of normal sperm (Matorras *et al.*, 2012; 2014; Ombelet, 2017; Farquhar *et al.*, 2018; Boomsma *et al.*, 2019). However, in several ART situations it is mandatory to use frozen-thawed sperm, a process that impairs sperm quality (Matorras *et al.*, 1996; Gómez-Torres *et al.*, 2017), so numerous strategies have been proposed to ameliorate the sperm quality and thus improve ART pregnancy rates or make it possible to perform less complex ART techniques.

Plasma rich in growth factors (PRGF) is an autologous platelet rich plasma (PRP) with proven efficacy in various clinical settings such as traumatology, maxillofacial surgery and plastic surgery (Borzini & Mazzucco, 2007; Sánchez *et al.*, 2008; Anitua *et al.*, 2015). In recent years, some reports have highlighted the role of PRP in the endometrium (Anitua *et al*., 2016; Farimani *et al*., 2019; Pantos *et al*., 2019).

PRGF is a safe, efficacious therapeutic approach obtained by extraction of a small amount of blood which is then centrifuged and activated to generate a plasma free of leucocytes which is enriched in proteins and growth factors (Anitua *et al*., 2012; Sheykhhasan & Seifalian, 2021). After activation, α-granules of platelets release hundreds of compounds that stimulate biological processes such as cell proliferation, recruitment and differentiation, chemotaxis, and angiogenesis (Anitua, 2001; Anitua *et al.*, 2016; Ferrari *et al.*, 2021).

A number of these molecules have been shown to improve sperm quality and function (Lee *et al.*, 2016). Thus TGF-β (transforming growth factor β) (Sharkey *et al.*, 2016), fibroblast growth factor (FGF) (Saucedo *et al*., 2015), vascular endothelial growth factor (VEGF) (Iyibozkurt *et al*., 2009) and serotonin (Jiménez-Trejo *et al.*, 2012) were found to enhance sperm motility (Iyibozkurt *et al*., 2009; Sharkey *et al.*, 2016), while zinc and calcium ions were seen to improve sperm capacitation and the acrosome reaction (Li *et al.*, 2016; Kerns *et al*., 2018). Moreover, the addition of nerve growth factor (NGF), insulin-like growth factor 1 (IGF-1), platelet-activating factor (PAF), ATP, zinc ions and superoxide dismutase as cryoprotectants have been reported to improve the quality of sperm after cryopreservation in animals (Padilha *et al*., 2012; Perumal *et al*., 2013; Kim *et al*., 2016) and humans (Rossi *et al*., 2001; Kotdawala *et al*., 2012; Saeednia *et al*., 2016). It has recently been reported that the addition of PRP to the cryoprotectant medium in normozoospermic samples increased sperm motility and viability after thawing (Yan *et al.*, 2021). PRP has been reported to improve sperm vitality and motility, and to decrease sperm DNA fragmentation and ROS levels (Bader *et al*., 2020).

For instance, exposure to FGF, a component of PRP, can increase FGFR phosphorylation levels of sperm flagella and activate extracellular signal-regulated kinase and protein kinase B signaling pathways, thereby promoting a significant increase in sperm progressive motility (Saucedo *et al*., 2015). It was also reported that VEGF (another PRP ingredient) ameliorates sperm motility parameters in a concentration-dependent manner *in vitro* (Iyibozkurt *et al*., 2009). Serotonin contained in PRP has also been shown to improve the curvilinear velocity of sperm components (Jiménez-Trejo *et al.*, 2012).

In addition, PRPs have shown an antioxidant effect on sperm (Bader *et al*., 2020; Yan *et al.*, 2021) as well as a buffering effect against osmotic shock (Lee *et al*., 2016).

The aim of our study was to assess whether the addition of PRGF to healthy sperm (no history of infertility and a total motile sperm count greater than 50 million in fresh or thawed) is safe, by evaluating the motility and vitality of the sperm.

## MATERIAL AND METHODS

### Population

The population of the study consisted of 44 sperm donors who attended our clinic over a six-month period and wished to participate in the study. All donors signed the corresponding informed consent form. Our study was approved by our Institutional Board (PI2015062) and was registered at trial registration (NCT02708537)

The sperm-donor selection protocol was previously described (Matorras *et al*., 2022). Briefly, this consisted of a medical and reproductive history with an investigation of drug intake and smoking habits, and a psychological interview. Physical examination, biometric measurement, blood analyses (general analyses, serologic tests, karyotype and systematic investigation of recessive diseases), sperm cultures, and spermiogram post-thawing sperm survival analysis were also performed (Matorras *et al*., 2022).

### Semen samples

Semen samples were collected into sterile plastic containers by masturbation following four-five days of sexual abstinence. After liquefaction, sperm analysis was performed. Ejaculates were managed according to the World Health Organization manual for examining and processing human semen (WHO, 2010). Concentrations of sperm and count of motile sperm (Matorras *et al*., 2022) were determined by a Makler counting chamber (Sefi Medical Instruments, Haifa, Israel) using standard clinical procedures. Sperm motility was evaluated according to WHO criteria (WHO, 2010). Sperm vitality was assessed with Sperm VitalStain (Nidacom, Mölndal, Sweden), which is a one-step vital staining technique containing eosin and nigrosin.

A drop (25µL) of semen was deposited in a clean tube, and a drop (25µl) of saline was added to allow good diffusion of the dye through the cytoplasmic membrane of the dead spermatozoa. It was mixed well and a spread was made on a slide. Under the microscope, a count was made of 200 spermatozoa and how many of them appeared unstained, i.e., those that were alive and had not been penetrated through the membrane by the dye. Results were expressed as a percentage and unstained sperm were classified as viable.

### PRGF preparation

On the day of seminal collection, nine mL of blood was collected to obtain PRGF. PRGF-Endoret^®^ technology was used, which employs calcium chloride (CaCl_2_) as a biocompatible platelet activator (Anitua, 2001). Blood samples for PRGF were immediately centrifuged at 580 g for eight minutes at room temperature in an Endoret System centrifuge (BTI Biotechnology Institute, S.L., Miñano, Spain). The whole plasma column above the buffy coat was harvested, taking care not to collect the layer containing leukocytes. Ten µl of CaCl_2_ (10 % wt/vol) were added per mL of plasma, the tube was gently turned upside down three-four times to ensure the correct distribution throughout the volume of plasma and placed in the Plasmaterm^®^ biological oven (BTI, Vitoria, Spain) at 37°C for at least 40 minutes, allowing fibrin to clot a pool of growth factors into the supernatant as it retracted. The resulting enriched-in-growth-factors supernatant (PRGF supernatant) was filtered through a polyester sulfone (PES) syringe filter and was ready to combine with the sperm samples.

### PRGF addition to sperm

In the first part of the study (n=15 sperm donors) we compared the influence of different PRGF dilutions (5%, 10%, 20% and 40%) on sperm motility, assessing them 30 minutes after the administration.

To do so, 400 µL was collected from the main fresh semen sample. Five aliquots of 80 µL were made: four were incubated in a Plasmaterm with different PRGF concentrations (5%, 10%, 20% and 40%) and one was used as a control. Samples were evaluated for motility 30 minutes after the addition of PRGF.

After analysis of semen motility and concentration, each of the aliquots was frozen by adding Freezing Medium (FUJIFILM Irvine Scientific, Tilburg, Netherlands). Each semen sample with autologous PRGF was diluted with equal volume 1:1 in test cryoprotectant medium glycerol-egg yolk-citrate freeze solution (20% egg yolk, 12% v/v glycerol, 10 µg/mL Gentamicin) and was cooled (2-8°C) for 40 minutes in a refrigerator. After the equilibration period, the mixtures were transferred into sterile cryovials. Finally, a -78°C CO_2_ dry ice mold was made and used to make several homogeneous pellets. After two-three minutes they were transferred to a 4.5 mL Nunc cryotube (Sigma Aldrich, Darmstadt, Germany) and completely immersed in liquid N_2_ (-196°C) for freezing. Then, subsequent thawing was performed and motility at 30 minutes was evaluated.

The second part of the study (n=29 sperm donors) contemplated a sequential study with the dilution at which there would have been a maximum effect on progressive motility in fresh semen. 20% of PRGF was added at different moments in fresh and in thawed sperm samples. A volume of 400 µL was collected from the main fresh semen sample; then, five 80-µL aliquots were prepared: four were mixed with 20% PRGF and incubated in a Plasmaterm, and one was used as a control without PRGF. Samples were evaluated at 15, 30 and 45 minutes after the addition of PRGF. After analysis of semen motility and concentration, each of the aliquots was frozen following the aforementioned protocol. After thawing the samples, concentration and motility were evaluated at 15, 30 and 45 minutes.

### Statistical analysis

**Figure S1:**
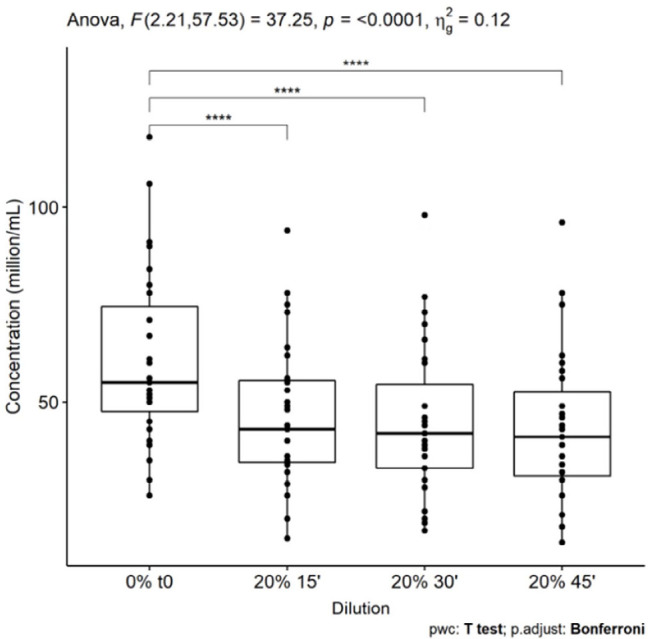
Boxplot of the ANOVA adjusted for multiple comparisons by Bonferroni test of the concentration of fresh samples.

**Figure S2:**
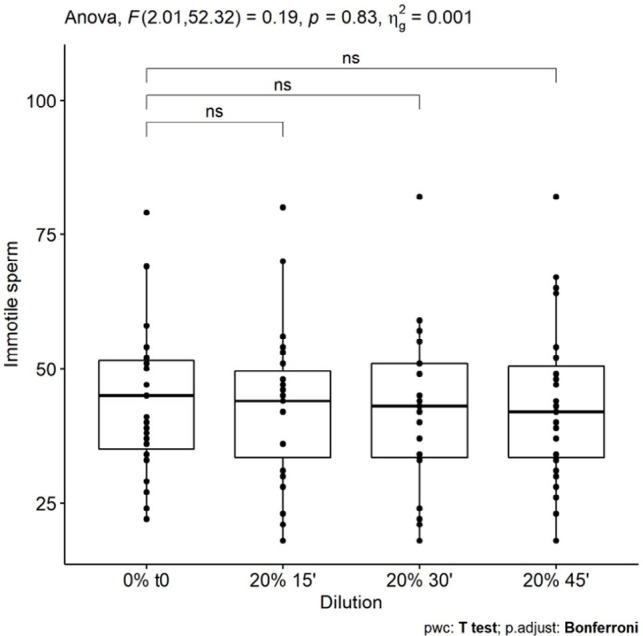
Boxplot of the ANOVA adjusted for multiple comparisons by Bonferroni test of the immotile sperm percentage of fresh samples.

**Figure S3:**
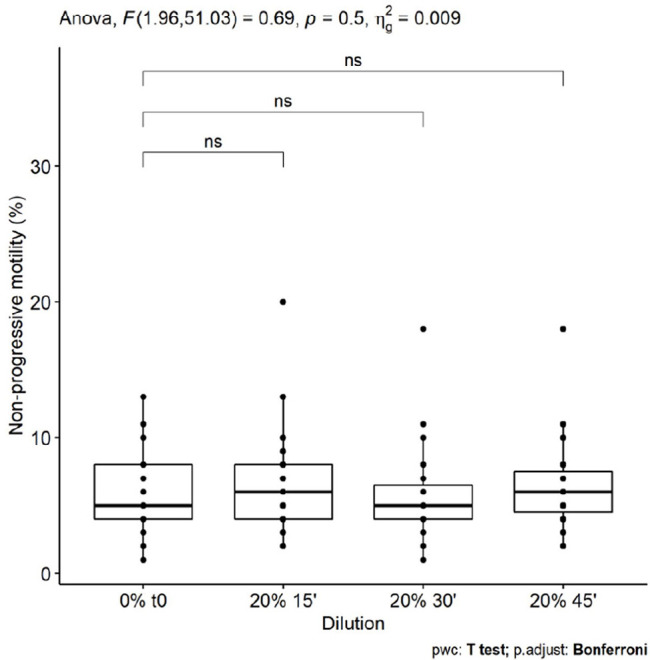
Boxplot of the ANOVA adjusted for multiple comparisons by Bonferroni test of the non-progressive sperm percentage of fresh samples.

**Figure S4:**
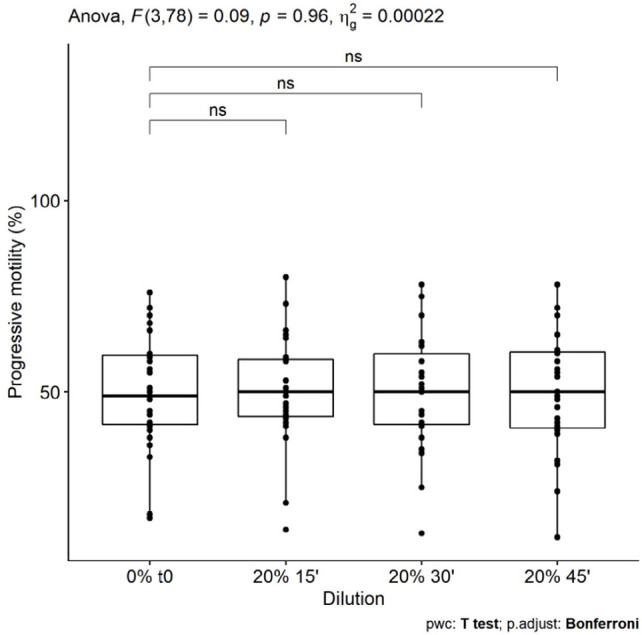
Boxplot of the ANOVA adjusted for multiple comparisons by Bonferroni test of the progressive sperm percentage of fresh samples.

**Figure S5:**
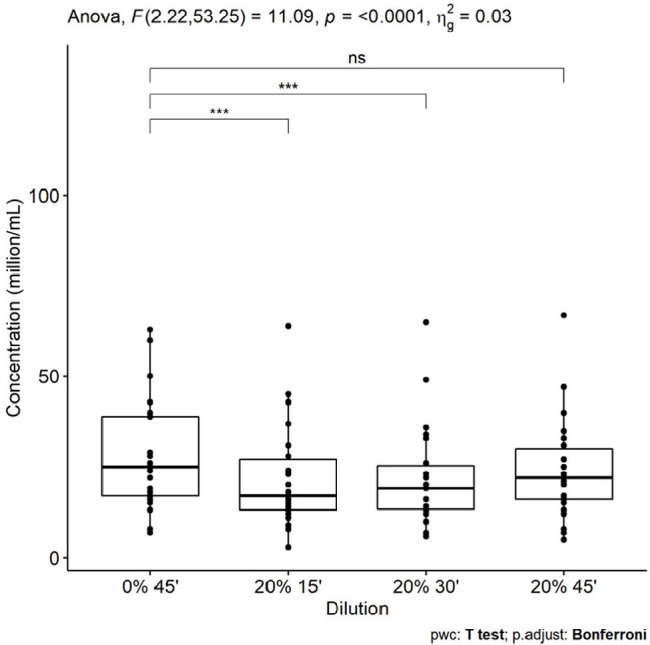
Boxplot of the ANOVA adjusted for multiple comparisons by Bonferroni test of the concentration of thawed samples.

**Figure S6:**
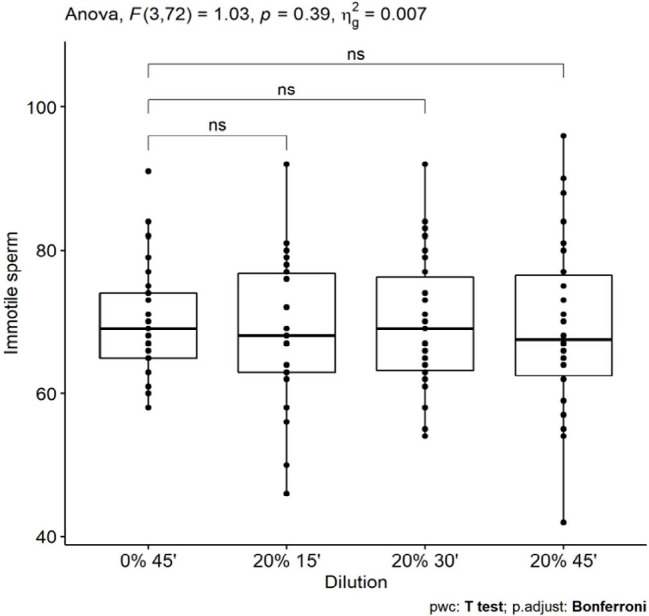
Boxplot of the ANOVA adjusted for multiple comparisons by Bonferroni test of the immotile sperm percentage of thawed samples.

**Figure S7:**
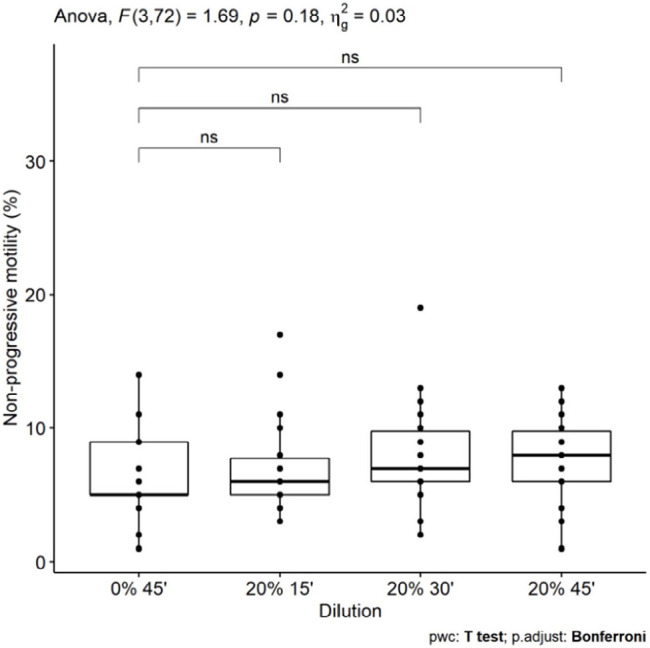
Boxplot of the ANOVA adjusted for multiple comparisons by Bonferroni test of the non-progressive sperm percentage of thawed samples.

**Figure S8:**
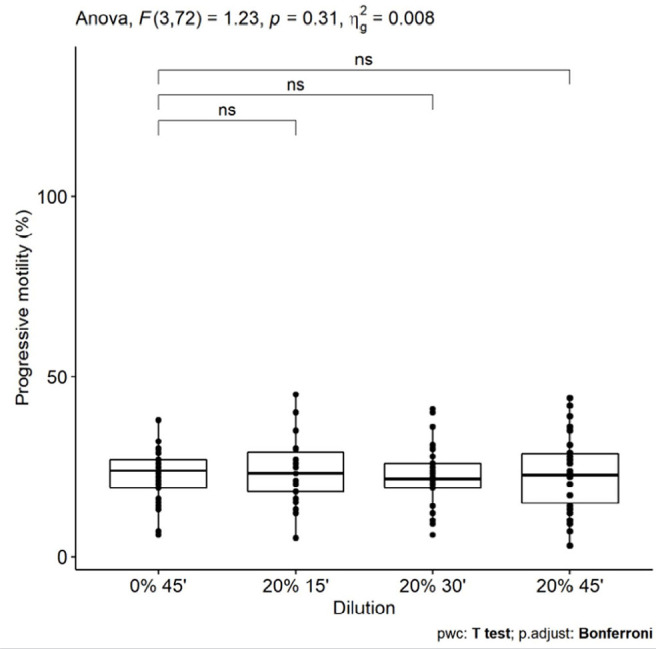
Boxplot of the ANOVA adjusted for multiple comparisons by Bonferroni test of the progressive sperm percentage of thawed samples.

Comparison of the effect of different PRGF dilutions on sperm samples assessed 15, 30 and 45 minutes after their administration was expressed as mean and standard deviation (SD). The normal distribution was assessed with the Shapiro-Wilk test and normal Q to Q plots. Mean of the differences between pairs with 95% confidence intervals were also presented. Differences between pre-post (15, 30 and 45 minutes) were assessed using repeated measures ANOVA adjusted for multiple comparisons by Bonferroni test. Boxplots were presented for these analyses (Figures S1-8, *link*).

Statistical analyses were performed using R (version 4.1.2): A language and environment for statistical computing. R Foundation for Statistical Computing, Vienna, Austria.

## RESULTS

### Patients

Mean age of donors in the study population was 22.52±3.52 years, mean weight 75.11±9.61 kg and 38.63% of them were smokers.

### Comparison of the effect of different PRGF dilutions on sperm motility after 30 minutes of incubation

There were no differences in sperm motility in fresh samples between the different dilutions of PRGF when assessed 30 minutes after administration, nor between them, nor when compared to the control group immediately prior to treatment (Table 1).

**Table 1:** Effect of PRGF on sperm parameters. Comparison of fresh and thawed samples 30 minutes after PRGF administration (t=30’) with an aliquot immediately before PRGF administration (t0). PRGF was added at different concentrations in dilutions of 0.5:10, 1:10, 2:10 and 4:10. Values are expressed as mean and standard deviation (SD).

Type	Motility classification	Control t0	t=30’			
		PRGF 0	PRGF 0.05	PRGF 0.1	PRGF 0.2	PRGF 0.4
Fresh samples	Progressive (%), Mean (SD)	49.27 (14.49)	44.71 (12.67)	46.77 (14.62)	49.07 (14.85)	47.99 (13.18)
	Non-progressive (%), Mean (SD)	6.10 (3.18)	5.95 (3.28)	5.45 (3.21)	6.26 (3.03)	5.89 (2.65)
	Immotile sperm (%), Mean (SD)	44.63 (14.06)	49.33 (15.95)	47.78 (16.27)	44.67 (14.99)	46.12 (15.17)
Thawed samples	Progressive (%), Mean (SD)	19.88 (6.64)	15.79 (6.13)	16.03 (6.35)	16.83 (7.87)	20.92 (6.75)
	Non-progressive (%), Mean (SD)	5.62 (2.70)	4.76 (2.3)	5.69 (2.68)	5.47 (2.22)	6.86 (2.49)
	Immotile sperm (%), Mean (SD)	74.5 (8.35)	79.45 (12.29)	78.27 (10.97)	77.7 (14.06)	72.22 (11.1)

PRGF, Plasma Rich Growth Factor.

No trend was observed between motility and PRGF dilution in linear regression analysis (r=0.000, *p*=0.99).

With respect to the thawed samples (Table 1), there were no significant differences, although there was a trend towards a progressive motility deterioration at 5% dilution, as well as a slight improvement at 40% dilution.

### Comparison of the effect of 20% PRGF dilution on sperm samples assessed 15, 30 and 45 minutes after administration

The sperm concentration in the fresh samples after the addition of PRGF, as expected because of dilution, was significantly lower at the three time points considered, compared to samples without PRGF, both at time 0 and 45 minutes (Table 2). The proportion of sperm with progressive motility, non-progressive motility and immotile sperm did not change compared with the samples without PRGF (Table 2).

**Table 2:** Effect of 20% PRGF on sperm parameters in fresh samples. Comparison of the samples 15, 30 and 45 minutes after PRGF administration with an aliquot immediately before PRGF administration and with another without PRGF 45 minutes later. Values are expressed as mean and standard deviation (SD). MD= Mean of the differences between pairs.

Parameter	Control t0	Post PRGF			Control t45’	MD Control t0 vs 15’ PRGF (95% CI)	MD Control t0 vs 30’ PRGF (95% CI)	MD Control t0 vs 45’ PRGF (95% CI)
		t=15’	t=30’	t=45’				
Concentration (million/mL), Mean (SD)	61.3 (22.7)	46.5 (18.7)	44.9 (19.6)	43.7 (19.0)	59.4 (22.9)	14.815 (10.803;18.827)	16.444 (12.779;20.11)	17.593 (13.264;21.921)
Progressive motility (%), Mean (SD)	50.0 (14.7)	50.1 (14.2)	49.6 (15.5)	49.9 (15.1)	47.7 (13.1)	-0.185 (-1.991;1.621)	0.407 (-2.009;2.824)	0.111 (-2.685;2.907)
Non-progressive motility (%), Mean (SD)	6.19 (3.23)	6.67 (3.68)	5.78 (3.40)	6.37 (3.08)	5.85 (2.63)	-0.481 (-1.68;0.717)	0.407 (-0.825;1.64)	-0.185 (-1.436;1.066)
Immotile sperm (%), Mean (SD)	43.5 (13.7)	43.0 (13.9)	42.3 (14.4)	43.5 (14.6)	45.3 (14.9)	0.481 (-1.528;2.491)	1.185 (-3.332;5.702)	0.000 (-3.041;3.041)
Vitality (%), Mean (SD)	55.5 (15.9)	-	-	-	54.2 (14.1)	-	-	-

PRGF, Plasma Rich Growth Factor.

As reported, in thawed samples a decrease in sperm concentration was observed after the addition of PRGF, again due to the dilution of the samples (Table 3). The progressive and non-progressive motility and the proportion of immotile spermatozoa did not change compared with the samples without PRGF.

**Table 3:** Effect of 20% PRGF on sperm parameters in thawed samples. Comparison of the samples with 20% PRGF 15, 30 and 45 minutes after thawing with a control aliquot without PRGF 45 minutes. Values are expressed as mean and standard deviation (SD). MD= Mean of the differences between pairs.

Parameter	Control 45’	Post PRGF			MD Control		
		t=15’	t=30’	t=45’	t45’vs 15’ PRGF (95% CI)	t45’ vs 30’ PRGF (95% CI)	t45’ vs 45’ PRGF (95% CI)
Concentration (million/mL), Mean (SD)	28.0 (14.9)	21.4 (13.7)	22.0 (13.4)	23.9 (13.3)	6.2 (3.426; 8.974)	5.64 (3.426; 8.974)	4.04 (3.426; 8.974)
Progressive motility (%), Mean (SD)	22.5 (7.52)	24.0 (9.32)	22.3 (8.84)	23.1 (10.8)	-1.96 (-4.569; 0.649)	0.08 (-4.569; 0.649)	-0.88 (-4.569; 0.649)
Non-progressive motility (%), Mean (SD)	6.36 (3.05)	7.23 (3.49)	7.92 (3.73)	7.50 (3.04)	-0.92 (-2.398; 0.558)	-1.48 (-2.398; 0.558)	-1.12 (-2.398; 0.558)
Immotile sperm (%), Mean (SD)	70.3 (7.88)	68.5 (10.6)	69.7 (9.77)	69.1 (12.5)	2.32 (-0.639; 5.279)	0.64 (-0.639; 5.279)	1.44 (-0.639; 5.279)
Vitality (%), Mean (SD)	30.8 (10.8)	29.6 (10.5)	30.0 (11.4)	28.2 (12.4)	0.88 (-1.246; 3.006)	0.36 (-1.246; 3.006)	2.84 (-1.246; 3.006)

PRGF, Plasma Rich Growth Factor.

**Figure 1:**
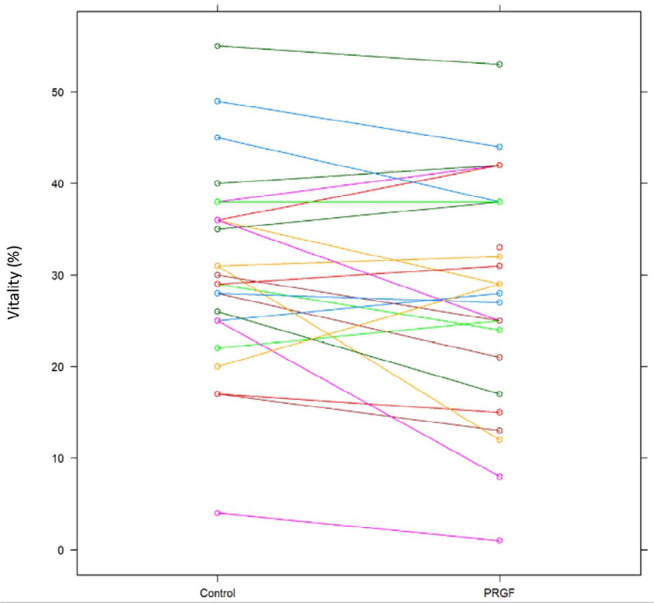
Changes in sperm vitality comparing thawed samples with 20% PRGF and without PRGF. Samples were analyzed 45 minutes after thawing and the vitality parameter is expressed in percentages (r=0.000, *p*=0.99).

Moreover, there were no changes in sperm vitality comparing samples with and without PRGF (Figure 1).

## DISCUSSION

Assisted reproductive techniques are very effective technologies to improve sperm quality and to increase/compensate the fertilizing ability of poor sperm. Usually, IVF/ICSI is needed with severely impaired sperm, whereas intrauterine insemination (IUI) is employed in slightly impaired or even normal sperm samples (Matorras *et al.*, 2012; 2014; Ombelet, 2017; Farquhar *et al.*, 2018; Boomsma *et al.*, 2019). In IUI a number of methods for sperm preparation/selection have been employed such as sperm washing (Boomsma *et al.*, 2019), swim-up (Karlström *et al*., 1991), gradient separation (Henkel & Schill, 2003), magnetic activated cell sorting (MACS) (Gil *et al*., 2013), microfluidics (Schuster *et al*., 2003; Suh *et al.*, 2005), electrophoresis zeta method (Chan *et al*., 2006) or hypo-osmotic swelling test (Suh *et al.*, 2005). Moreover, there are controversial reports regarding the administration to the sperm sample of different compounds, such as antioxidants (Griveau & Le Lannou, 1994), caffeine (Barkay *et al*., 1977), pentoxifylline (Stanic *et al.*, 2002), platelet activating factor (Wild & Roudebush, 2001; Matorras & Pijoan, 2002) or papaverine (Ibis *et al*., 2021). Another option is for the male to take different antioxidants (Smits *et al.*, 2019), vitamin E (Matorras *et al*., 2020) or micronutrients (Lipovac *et al*., 2016).

Furthermore, a number of compounds have been tested to protect sperm from freezing or to improve sperm after that process, such as the addition of reduced glutathione (GSH) (Gadea *et al*., 2011), curcumin (Nur Karakus *et al*., 2021), L- prolin (Moradi *et al*., 2022), alpha lipoic acid (Shaygannia *et al.,* 2020), pentoxifylline (Esteves *et al*., 2007) and theophylline (Ebner *et al*., 2011). Recently, autologous platelet-rich plasma has arisen as a good supplement in the process of cryopreservation of human or animal spermatozoa, offering a protective effect (Alcay *et al*., 2021; Yan *et al*., 2021).

There are some controversial reports regarding platelet derivatives and sperm motility. It has been reported that exogenous platelet-activating factor (ePAF) was associated with an increase in sperm motility in IUI samples (Alcay *et al*., 2021). The addition of platelet-activating factor was reported to improve IUI outcome (Wild & Roudebush, 2001; Roudebush *et al*., 2004), although the early report (Wild & Roudebush, 2001) lacked statistical significance (Matorras & Pijoan, 2002).

PRGF has been tested in several medical conditions. Among others, beneficial effects are well known in traumatology, dentistry and ophthalmology. In recent years, PRP has been used in reproductive medicine, mainly for endometrial growth (Anitua *et al.*, 2016) and ovarian rejuvenation (Atkinson *et al*., 2021). Many components present in PRGF have been reported to increase sperm motility when administered separately, such as TGF- β (Sharkey *et al.*, 2016), FGF (Saucedo *et al*., 2015), VEGF (Iyibozkurt *et al*., 2009) and serotonin (Jiménez-Trejo *et al*., 2012). When administered as cryoprotectants, other components like NGF, IGF-1, platelet-activating factor, ATP, zinc ions and superoxide dismutase, have been reported to improve the quality of sperm after cryopreservation in animals (Padilha *et al*., 2012; Perumal *et al*., 2013; Kim *et al*., 2016) and humans (Rossi *et al*., 2001; Kotdawala *et al*., 2012; Saeednia *et al*., 2016).

Platelet rich plasma (PRP) has been shown to improve motility and morphology in ram sperm (Hernández-Corredor *et al*., 2020). Furthermore, PRP administration to sperm confers protection against oxidative stress. In a recent report, the addition of 5% PRP to sperm specimens before freezing improved sperm progressive motility, viability and membrane integrity, while in another study 2% PRP treatment enhanced sperm parameters and prevented cell death in H_2_O_2_-exposed spermatozoa as compared to freshly collected semen (Bader *et al*., 2020; Yan *et al*., 2021).

Our experiment adding 20% PRGF did not influence sperm motility in fresh semen samples or in thawed samples, nor were there differences in sperm vitality after thawing. Moreover, we did not find any differences with the other dilutions tested (5%, 10%, 40%). The effect of PRGF on sperm samples was probably not detected because of the dilution factor. A recent study including 100 sperm samples showed that 2% was the optimal dose of PRP for providing substantial effects (Hamdan *et al*., 2021). The administration of 2% PRP to semen samples significantly improved human sperm motility (Hamdan *et al*., 2021). In this sense, further research modifying the PRGF dose should be considered to avoid the dilution of sperm specimens.

Furthermore, concerning the discrepancies of our work with previous studies (Bader *et al.*, 2020), we should highlight the following differences: we used PRGF as a supplementation to the sperm samples (either fresh or thawed), whereas in one previous study, platelet rich plasma (PRP) was used only in fresh specimens (Bader *et al.*, 2020) and, in the other, PRP was used as a supplementary cryoprotectant before freezing (Yan *et al*., 2021). Moreover, we assessed sperm parameters 15, 30 and 45 minutes after PRGF administration while in another study PRP samples were evaluated 24 hours later and in the other immediately after thawing (Li *et al.*, 2016). Previous reports concerning the best PRGF dilution are controversial. In one report, 2% PRP sperm motility increased, did not change with 5% and with 10% was impaired (Bader *et al.*, 2020). However, in another study motility improved only with 5% PRP, but not with 2% and 10% PRP (Li *et al.*, 2016). In the concentration range we studied of 5-40%, we observed no effect on motility in fresh or thawed samples, nor in vitality in thawed samples. A recently presented work showed that incubation of fresh semen samples with PRP for 1 hour produced significantly better quality in terms of concentration, motility, progressive motility, morphology and percentage of sperm, with good fertilization in 40 males (Angellee *et al*., 2021).

## CONCLUSION

Our study has shown the absence of any negative effect of administering PRGF on fresh or thawed sperm samples from healthy donors. This is important, as it will prepare the ground for assessing the effect of PRGF administration in subnormal sperm samples. In fact, our study population consisted of young, healthy sperm donors (mean age 22 years) compared to men in the third decade (mean age 35 years) with normal sperm attending an IVF clinic (Bader *et al.*, 2020) and to normozoospermic men consulting for fertility evaluation (Yan *et al*., 2021). Furthermore, the most relevant marker of sperm quality, pregnancy rate, was not assessed in our study.

More studies focusing on subnormal sperm samples, analyzing different PRGF concentrations and increasing the number of study variables are needed.
